# Supplementary material for: Genetic Diversity of Potyviruses Associated with Tulip Breaking Syndrome
Source: Plants (Basel). 2020 Dec 19;9(12):1807. doi: 10.3390/plants9121807 (PMC7766433; doi:10.3390/plants9121807)
Supplement: Supplementary file 1 [file plants-09-01807-s001.zip › Table S1.docx]

S1 Table. Tulip samples collected in 2017 and 2018 growing seasons

| **Number** | **Group^1^** | **Cultivar** | **Location** | **GPS coordinates** | **Collection date** |
| --- | --- | --- | --- | --- | --- |
| 1 | Darwin-hybrid | Apeldoorn | Gödöllő | N47.609981 E19.344069 | 18/04/2017 |
| 2 | Viridiflora | Groenland | Gödöllő | N47.609981 E19.344069 | 06/05/2017 |
| 3 | Rembrandt | Absalon (1780) | Budapest | N47.514508 E19.010534 | 15/01/2018 |
| 4 | Rembrandt | Zomerschoon (1620) | Budapest | N47.514508 E19.010534 | 24/01/2018 |
| 5 | Rembrandt | Insulinde (<1915) | Budapest | N47.514508 E19.010534 | 24/01/2018 |
| 6 | Fringed/Crispa | Barbados | Nyársapát | N47.087373 E19.780381 | 25/03/2018 |
| 7 | Darwin-hybrid | Apeldoorn | Püspökladány | N47.319997 E21.107208 | 01/04/2018 |
| 8 | Fringed/Crispa | Lambada | Budapest | N47.480482 E19.038670 | 16/04/2018 |
| 9 | Fringed/Crispa | Crystal Beauty | Budapest | N47.480482 E19.038670 | 16/04/2018 |
| 10 | Darwin-hybrid | Apeldoorn | Balatonalmádi | N47.060221 E18.025412 | 16/04/2018 |
| 11 | Darwin-hybrid | Apeldoorn | Deszk | N46.220471 E20.244462 | 18/04/2018 |
| 12 | Darwin-hybrid | Apeldoorn | Murony | N46.761155 E21.042277 | 18/04/2018 |
| 13 | Darwin-hybrid | Apeldoorn | Törökszentmiklós | N47.174697 E20.381891 | 22/04/2018 |
| 14 | Darwin-hybrid | Apeldoorn | Miskolc | N48.100427 E20.703571 | 19/04/2018 |
| 15 | Darwin-hybrid | Apeldoorn | Felsőtárkány | N47.965981 E20.406040 | 19/04/2018 |
| 16 | Darwin-hybrid | Apeldoorn | Bátonyterenye | N47.979458 E19.812472 | 19/04/2018 |
| 17 | Darwin-hybrid | Apeldoorn | Nemesbőd | N47.267700 E16.735999 | 20/042018 |
| 18 | Darwin-hybrid | Apeldoorn | Győrújbarát | N47.619911 E17.642959 | 20/042018 |
| 19 | Darwin-hybrid | Apeldoorn | Komárom | N47.737643 E18.132819 | 20/042018 |
| 20 | Darwin-hybrid | Apeldoorn | Szada | N47.636704 E19.326267 | 21/042018 |
| 21 | Triumph | Oscar | Szada | N47.636704 E19.326267 | 21/042018 |
| 22 | Triumph | First Class | Szada | N47.636704 E19.326267 | 21/042018 |
| 23 | Double Late | Blue Diamond | Szada | N47.636704 E19.326267 | 21/042018 |
| 24 | Parrot | Blue Parrot | Szada | N47.636704 E19.326267 | 21/042018 |
| 25 | Darwin-hybrid | Gudoshnik | Szada | N47.636704 E19.326267 | 21/042018 |
| 26 | Fosteriana | Purissima | Szada | N47.636704 E19.326267 | 21/042018 |
| 27 | Parrot | Texas Gold | Szada | N47.636704 E19.326267 | 21/042018 |
| 28 | Darwin-hybrid | Apeldoorn | Gárdony | N47. 201102 E18.619450 | 21/042018 |
| 29 | Darwin-hybrid | Apeldoorn | Tolna-Mözs | N46.411774 E18.753034 | 22/04/2018 |
| 30 | Darwin-hybrid | Apeldoorn | Pécs | N46.051745 E18.224481 | 22/04/2018 |
| 31 | Darwin-hybrid | Apeldoorn | Zalaegerszeg | N46.841650 E16.878243 | 22/04/2018 |
| 32 | Lily-flowered | Claudia | Mátraszentimre | N47.909846 E19.876930 | 02/05/2018. |

^1^ According to van Scheepen [30].
