# Supplementary material for: Genetic Diversity of Potyviruses Associated with Tulip Breaking Syndrome
Source: Plants (Basel). 2020 Dec 19;9(12):1807. doi: 10.3390/plants9121807 (PMC7766433; doi:10.3390/plants9121807)
Supplement: Supplementary file 1 [file plants-09-01807-s001.zip › Table S2.docx]

Table S2. Results of ELISA tests, the identified viruses, their isolates and accession numbers

| **Number** | **acc. #** | **Group^1^** | **Cultivar** | **Virus acronym^2^** | **ratio of ELISA absorbance means**  **(sample/negative control)** |
| --- | --- | --- | --- | --- | --- |
| 1 | MF983709 | Darwin-hybrid | Apeldoorn | LMoV | 4.000 |
| 2 | MF983710 | Viridiflora | Groenland | TBV | 5.000 |
| 3 | MK368780 | Rembrandt | Absalon (1780) | ReTBV | 13.400 |
| 4 | MK368781 | Rembrandt | Zomerschoon (1620) | ReTBV | 4.200 |
| 5 | MK368782 | Rembrandt | Insulinde (<1915) | ReTBV | 11.600 |
| 6 | MK368783 | Fringed/Crispa | Barbados | TBV | 29.642 |
| 7 | MK368784 | Darwin-hybrid | Apeldoorn | LMoV | 7.605 |
| 8 | MK368785 | Fringed/Crispa | Lambada | TBV | 51.164 |
| 9 | MK368786 | Fringed/Crispa | Crystal Beauty | TBV | 30.200 |
| 10 | MK368787 | Darwin-hybrid | Apeldoorn | TBV | 50.970 |
| 11 | MK368788 | Darwin-hybrid | Apeldoorn | LMoV | 5.000 |
| 12 | MK368789 | Darwin-hybrid | Apeldoorn | TBV | 11.000 |
| 13 | MK368790 | Darwin-hybrid | Apeldoorn | LMoV | 10.200 |
| 14 | MK368791 | Darwin-hybrid | Apeldoorn | TBV | 15.600 |
| 15 | MK368792 | Darwin-hybrid | Apeldoorn | LMoV | 22.200 |
| 16 | MK368793 | Darwin-hybrid | Apeldoorn | LMoV | 6.200 |
| 17 | MK368794 | Darwin-hybrid | Apeldoorn | LMoV | 6.600 |
| 18 | MK368795 | Darwin-hybrid | Apeldoorn | TBV | 4.600 |
| 19 | MK368796 | Darwin-hybrid | Apeldoorn | TBV | 11.030 |
| 20 | MK368797 | Darwin-hybrid | Apeldoorn | TBV | 4.700 |
| 21 | MK368798 | Triumph | Oscar | TBV | 37.400 |
| 22 | MK368799 | Triumph | First Class | TBV | 26.400 |
| 23 | MK368800 | Double Late | Blue Diamond | TBV | 50.910 |
| 24 | MK368801 | Parrot | Blue Parrot | LMoV | 51.313 |
| 25 | MK368802 | Darwin-hybrid | Gudoshnik | LMoV | 51.746 |
| 26 | MK368803 | Fosteriana | Purissima | LMoV | 8.800 |
| 27 | MK368804 | Parrot | Texas Gold | LMoV | 27.500 |
| 28 | MK368805 | Darwin-hybrid | Apeldoorn | TBV | 3.900 |
| 29 | MK368806 | Darwin-hybrid | Apeldoorn | LMoV | 7.800 |
| 30 | MK368807 | Darwin-hybrid | Apeldoorn | TBV | 3.500 |
| 31 | MK368808 | Darwin-hybrid | Apeldoorn | TBV | 5.300 |
| 32 | MK368809 | Lily-flowered | Claudia | LMoV | 68.100 |

^1^ According to van Scheepen [30].

^2^ Virus acronyms are used in accordance with Brunt [18].
