# Supplementary material for: Genetic Diversity of Potyviruses Associated with Tulip Breaking Syndrome
Source: Plants (Basel). 2020 Dec 19;9(12):1807. doi: 10.3390/plants9121807 (PMC7766433; doi:10.3390/plants9121807)
Supplement: Supplementary file 1 [file plants-09-01807-s001.zip › Table S3.docx]

S4 Table. Amino acid sequences from positions P4 to P1’ at the NIb/CP cleavage site

| Accession nr.: | Acronym | NIb/CP cleavage site |
| --- | --- | --- |
| AB078007 | LMoV | not available |
| AB090385 | LMoV | not available |
| EU267778 | LMoV | not available |
| KJ561805 | LMoV | not available |
| MF781080 | LMoV | not available |
| AB053256 | LMoV | VAFQ/A |
| AF531458 | LMoV | VAFQ/A |
| AJ310203 | LMoV | VAFQ/A |
| FJ618539 | LMoV | VAFQ/A |
| JN127341 | LMoV | VAFQ/A |
| KF553658 | LMoV | VAFQ/A |
| MF983709 | LMoV | VAFQ/A |
| MH360239 | LMoV | VAFQ/A |
| MK368784 | LMoV | VAFQ/A |
| MK368788 | LMoV | VAFQ/A |
| MK368790 | LMoV | VAFQ/A |
| MK368792 | LMoV | VAFQ/A |
| MK368793 | LMoV | VAFQ/A |
| MK368794 | LMoV | VAFQ/A |
| MK368801 | LMoV | VAFQ/A |
| MK368802 | LMoV | VAFQ/A |
| MK368803 | LMoV | VAFQ/A |
| MK368804 | LMoV | VAFQ/A |
| MK368806 | LMoV | VAFQ/A |
| MK368809 | LMoV | VAFQ/A |
| S44147 | LMoV | VAFQ/A |
| AB674535 | ReTBV | VIFQ/A |
| JN127335 | ReTBV | VIFQ/A |
| MG637051 | ReTBV | VIFQ/A |
| MK368780 | ReTBV | VIFQ/A |
| MK368782 | ReTBV | VIFQ/A |
| MK368781 | ReTBV | VILQ/A |
| KF442403 | TBV | VQFQ/A |
| KT923168 | TBV | VQFQ/A |
| MF983710 | TBV | VQFQ/A |
| MK368783 | TBV | VQFQ/A |
| MK368785 | TBV | VQFQ/A |
| MK368786 | TBV | VQFQ/A |
| MK368787 | TBV | VQFQ/A |
| MK368789 | TBV | VQFQ/A |
| MK368791 | TBV | VQFQ/A |
| MK368795 | TBV | VQFQ/A |
| MK368796 | TBV | VQFQ/A |
| MK368797 | TBV | VQFQ/A |
| MK368798 | TBV | VQFQ/A |
| MK368799 | TBV | VQFQ/A |
| MK368800 | TBV | VQFQ/A |
| MK368805 | TBV | VQFQ/A |
| MK368807 | TBV | VQFQ/A |
| MK368808 | TBV | VQFQ/A |
| X63630 | TBV | VQFQ/A |
| NC_001555 | TEV | LYFQ/S |
