# Supplementary figures and images for: Genetic Diversity of Potyviruses Associated with Tulip Breaking Syndrome
Source: Plants (Basel). 2020 Dec 19;9(12):1807. doi: 10.3390/plants9121807 (PMC7766433; doi:10.3390/plants9121807)

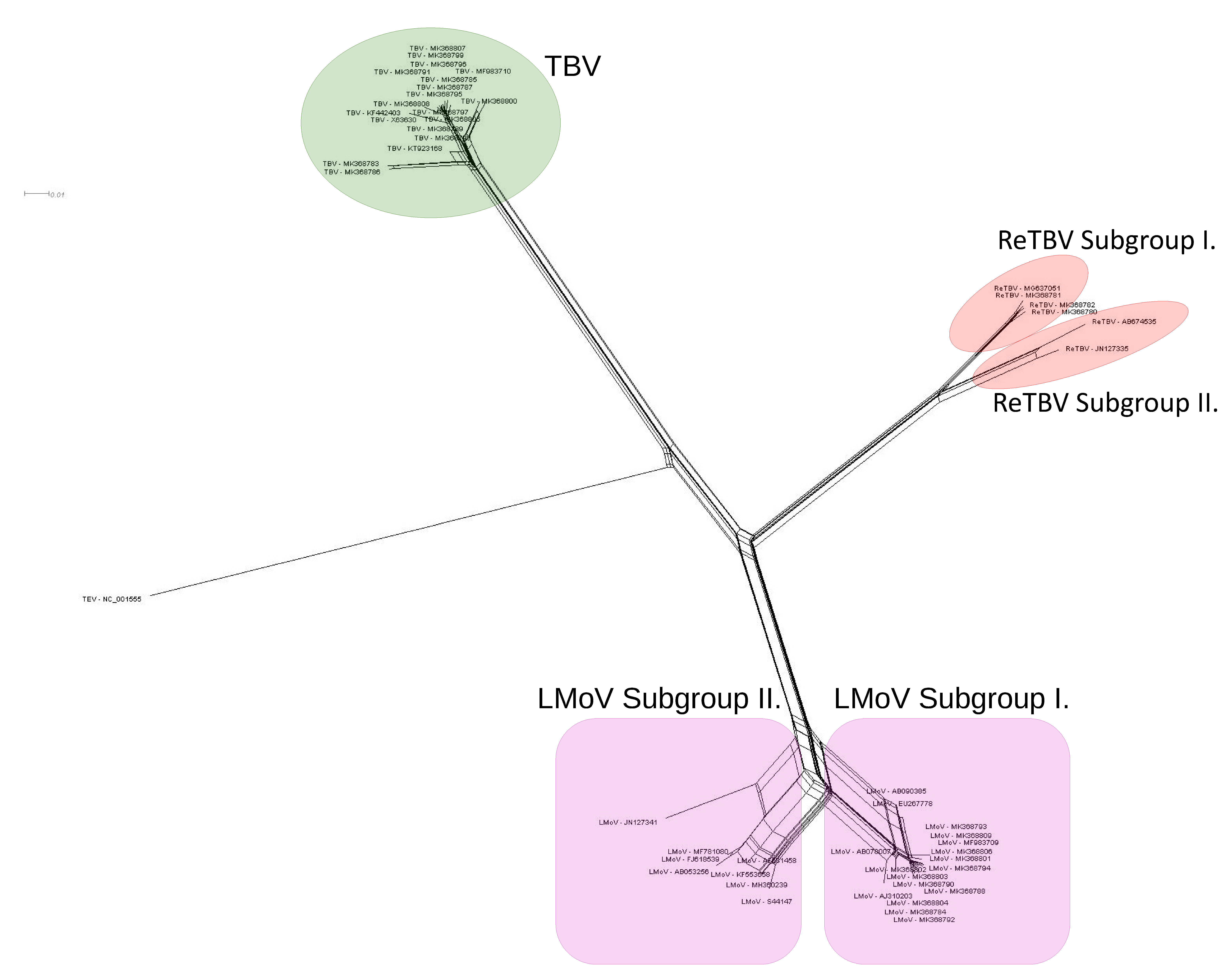

Supplement: Supplementary file 1 [file plants-09-01807-s001.zip › Fig S1.jpg]
